# Cycling or Swapping in Spondyloarthritis? Current Knowledge

**DOI:** 10.31138/mjr.041025.eor

**Published:** 2026-01-08

**Authors:** Sofia Karagianni, Niki Kyriazi, Nikolaos Michalakeas, Konstantinos D. Vassilakis, George E. Fragoulis

**Affiliations:** 1 Joint Academic Rheumatology Program, First Department of Propaedeutic and Internal Medicine, National and Kapodistrian University of Athens, Athens, Greece;; 2 Rheumatology Department, “Evangelismos” Hospital, Athens, Greece;; 3 University of Glasgow, School of Infection and Immunity, Glasgow, United Kingdom

**Keywords:** psoriatic arthritis, axial spondyloarthritis, cycling, swapping

## Abstract

Psoriatic arthritis (PsA) and axial Spondyloarthritis (axSpA) share some common therapeutic options, namely TNF-inhibitors, anti-IL-17 and JAK-inhibitors. Despite the wealth of choices, often patients do not respond to the first b/tsDMARDs, but they have to switch to another one, either of the same therapeutic category (cycling) or of another (swapping). Which of the abovementioned strategies is better is ill-defined thus far. In this narrative review, we aimed to critically present the relevant literature. PubMed was searched from inception to June 2025 using the following keywords: “Psoriatic arthritis” OR “axial spondyloarthritis” OR “ankylosing spondylitis” OR “non-radiographic axial spondyloarthritis” AND “cycling” OR “swapping” or “switching”. Only studies enrolling adult population were included, while non-English literature was excluded. For PsA, it seems that both cycling and swapping are equally effective, although this might not be the case for all b/tsDMARDs and/or for all patients (e.g. there might be gender differences). For AxSpA, switching to a second TNFi is reasonable, however, treatment effectiveness generally declines. For secondary non-responders, switching to a different mechanism of action seems to be valid strategy. Conclusively, both cycling and swapping strategies appear to be equally successful in PsA and AxSpA. Characteristics, like gender, might affect the efficacy of the one versus the other strategy. In both cases, assessment of the risk factors is warranted.

## INTRODUCTION

The concept of spondyloarthritides (SpA) encompasses a group of rheumatic diseases that share common characteristics including axial spondyloarthritis (ax-SpA), psoriatic arthritis (PsA), arthritis/spondylitis related to inflammatory bowel disease (IBD) and reactive arthritis.

PsA is a chronic inflammatory disease,^1^ which is estimated to affect 0.1–1.0% of the general population and approximately 20–30% of individuals with psoriasis (PsO).^2^ It is a multifaceted condition, characterised by peripheral arthritis, along with other musculoskeletal symptoms, such as enthesitis, dactylitis, and axial disease.^3^ Additionally, PsA may be linked to other coexisting conditions, including cardiometabolic disorders and mental health issues.^4–6^ Numerous therapeutic options are available for the management of PsA. These include conventional synthetic (cs) disease- modifying antirheumatic drugs (DMARDs), as well as biological (b) DMARDs targeting tumour necrosis factor (TNF), Interleukin (IL)- 23 and IL- 17 and targeted synthetic (ts) DMARDs that inhibit Janus kinases (JAKs) or phosphodiesterase 4 (PDE4).^7^ However, achieving low or minimal disease activity (LDA/MDA),^2^ remains challenging. Between 40 and 80% of people with PsA either discontinue or switch their b/tsDMARD therapy within two years and only 30% of patients achieve MDA in the first year of treatment.^8^ This inevitably leads to cycling or swapping of b/tsDMARDs, which means change to a b/tsDMARDs of the same or a different category, respectively. Nevertheless, there is no clear consensus whether to cycle or swap after an inadequate response or intolerance to a bDMARD or a JAK inhibitor (JAKi). This is also reflected in the recent EULAR recommendations for the management of PsA.^9^

AxSpA is a chronic inflammatory condition that predominantly affects spine and sacroiliac joints, leading to chronic back pain, spinal stiffness, and impaired quality of life. Patients may also present with peripheral manifestations (arthritis, enthesitis, dactylitis) and extra-musculoskeletal symptoms (uveitis, IBD). The term encompasses both radiographic (established radiographic sacroiliac joints damage) also known as ankylosing spondylitis (AS) and non-radiographic ax-SpA.^10^

In axSpA after inadequate response to non-steroidal anti-inflammatory drugs (NSAIDs), physical exercise and/or physiotherapy, b/ts DMARDs are initiated (such as a TNFi or an IL-17i). If therapy with the first TNFi fails, switching to an alternative TNFi or IL-17i or JAKi is recommended, based on the latest recommendations of Assessment of SpondyloArthritis international Society (ASAS)- EULAR.^11^ TNFi have shown remarkable efficacy in the treatment of SpA. However, up to 30% of patients may discontinue therapy mainly due to loss of efficacy or adverse events.^12^ There is limited data regarding most suitable sequence after failure of the first TNFi. Although 64–68% of patients respond to a second TNFi, primary non-response of two TNFi strongly predicts ineffectiveness of the third.^13^

Herein, we aim to review the current knowledge in cycling and swapping strategies in PsA and AxSpA. We searched PubMed from inception to June 2025 using the following keywords: “Psoriatic arthritis” OR “axial spondyloarthritis” OR “ankylosing spondylitis” OR “non-radiographic axial spondyloarthritis” AND “cycling” OR “swapping” or “switching”. Only studies enrolling adult population were included, while non-English literature was excluded. We adhered to the recommendations by Gasparyan et al. on writing a narrative review.^14^ Initially, we present studies examining cycling (same therapeutic category) and next those which refer to swapping (different therapeutic category) strategies; finally, we present studies which compare these two strategies (cycling Vs swapping). PsA and AxSpA are presented sequentially.

## CYCLING IN PSA

### TNF-inhibitors

**Figure 1.**
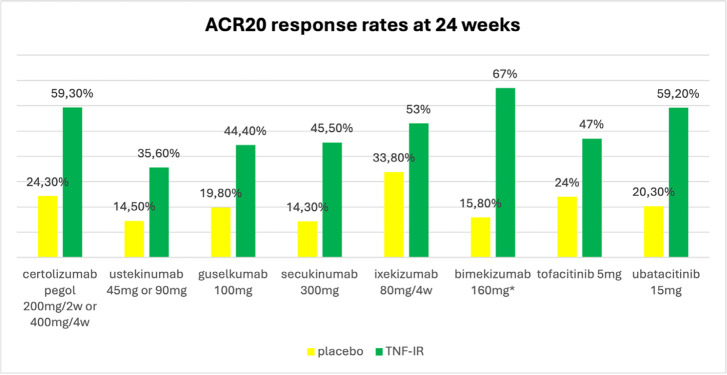
Proportions of patients achieving American College of Rheumatology 20% improvement criteria (ACR20) response rates at week 24. *TNF-IR: TNF inadequate responders, ** ACR20 response at week 16, ^ ACR20 response at week 12. Created by the authors using Microsoft Excel. Elements are authentic and not adapted from elsewhere. The comparisons shown are indirect with high risk of bias. The homogeneity of the population between studies cannot be assessed.

Cycling between TNFi, has been proven efficient for the treatment of PsA. There are several studies showing that cycling between TNFi is a valid and effective strategy.^15^ A longitudinal retrospective study,^16^ assessed the persistence and effectiveness of golimumab as a second TNFi. Data were collected from 20 centers in Spain. 210 patients, from whom 79 had PsA, were included. Treatment with golimumab was effective, with mean (SD) baseline Disease Activity Score [DAS] reducing from 4.0 (1.3) to 2.5 (1.2) at month 3 and to 2.2 (1.3) at year 1. Certolizumab pegol has also demonstrated efficacy in a phase III, randomised, double-blind, placebo-controlled trial conducted over 24 weeks.^17,18^ A total of 409 patients were enrolled, of whom 19.8% had prior exposure to TNFi. Participants were randomized in a 1:1:1 ratio to receive placebo, certolizumab pegol 200 mg every two weeks (Q2W), or certolizumab pegol 400 mg every four weeks (Q4W). At week 24, American College of Rheumatology 20% response criteria [ACR20] was achieved by 59.3% of TNFi experienced patients (**Figure 1**) and 60.3% of TNFi naïve patients vs 24.3% of placebo patients (p< 0.05 vs placebo). MDA was attained in 34.1% and 33.3% of patients in the Q2W and Q4W groups, respectively, versus 5.9% in the placebo group (p < 0.001).

On the other hand, it is also well known that response rates to TNFi are significantly lower with the second TNFi compared to the first.^15^ This is also evident in a Norwegian longitudinal observational study,^19^ where 439 patients with PsA were included. Of these, 344 were initiating their first TNFi, while 95 had switched to a second TNFi. Three-month responses were compared between the two groups. Patients with prior TNFi exposure exhibited significantly poorer outcomes than TNFi naïve patients (ACR50 response: 22.5% vs 40.0%, p=0.05 DAS28 remission: 28.2% vs 54.1%, p=0.006).

### IL-17 inhibitors

The efficacy of cycling between IL-17i has also been investigated, to a lesser extent though. Hitherto, it seems that cycling between these therapeutic regimes is also effective. A multicenter case series by Panagiotopoulos et al.^20^ evaluated the effectiveness of cycling (with or without having other b/tsDMARDs in between) from one IL-17i, secukinumab (SEC), to another, ixekizumab (IXE), in patients with PsA, who had an inadequate response to secukinumab. 24 PsA patients who received IXE either immediately or after ≥ 1 interposed b-DMARD were followed for an average of 9.6 months. Treatment with IXE proved effective, with Disease Activity in PSoriatic Arthritis (DAPSA) scores for peripheral arthritis significantly decreasing from 22.8 to 13.6 (p = 0.001), and psoriasis involvement improving as Body Surface Area (BSA) reduced from 8.7% to 2.4% (p=0.001). Among patients with axial disease, 50% demonstrated a clinically meaningful improvement (Δ Ankylosing Spondylitis Disease Activity Score [ΔASDAS] ≥ 1.1).

In another retrospective, multi-center observational study,^21^ Berman et al. aimed to evaluate the clinical effectiveness of ixekizumab in PsA patients who previously failed treatment with secukinumab. 23 PsA patients with a history of treatment with secukinumab, further treated with ixekizumab for a minimum of 3 months, were included. 83% of patients achieved a primary response to ixekizumab, with the overall treatment duration during follow-up being approximately 14 months (IQR 10–20.5). DAPSA, Tender Joint Count (TJC) and Simplified Disease Activity Index (SDAI) were significantly lower at 6 and 12 months (DAPSA decreased by 1.5–2 levels at 6 months (p=0.018) and 1–1.5 at 12 months (p=0.031); TJC by −2.16 (p=0.025) and −1.69 (p=0.022); SDAI by −10.13 (p=0.003) and −12.2 (p=0.0002), respectively).

### IL-23 and IL12/23 inhibitors

IL-23p19 inhibitors (IL-23p19i) (guselkumab, risankizumab, tildrakizumab), as well as IL-12/23 inhibitors (IL-12/23) (Ustekinumab) have demonstrated efficacy and safety in the management of PsA, especially when psoriasis is a predominant manifestation.^22,23^ Nonetheless, the therapeutic strategy of cycling between IL-23i in PsA remains unexplored. Limited evidence can be lent from the effectiveness of IL-23i cycling in the treatment of psoriasis. A recent retrospective analysis of a PsO patient registry^24^ evaluated 169 individuals who received guselkumab following primary or secondary failure of ustekinumab. After three years of treatment, clinically significant improvements were observed, with 88.4%, 55.8%, and 32.6% of patients achieving Psoriasis Area and Severity Index (PASI) 75, PASI 90, and PASI 100 responses, respectively. These findings suggest potential benefit in cycling between IL-23 and IL12/23 inhibitors.

Collectively, one could say that cycling between TNFi and IL-17i is an effective strategy, while more data are needed for IL-23i.

## SWAPPING IN PSA

Swapping in PsA has also been investigated, proving that alternating between medications with different modes of action is a valid strategy. Swapping from TNFi to IL-23i, and IL-17i have been investigated, though data for other strategies are very limited.

### TNFi to ustekinumab

The phase III PSUMMIT II trial, a multicentre, placebo-controlled study, involved 312 patients with PsA who had previously been treated with conventional therapies and/or TNFi.^25^ Participants were randomly assigned to receive ustekinumab at doses of either 45 mg or 90 mg at weeks 0, 4, and then every 12 weeks, or a placebo at weeks 0, 4, and 16, followed by a switch to ustekinumab 45 mg at weeks 24, 28, and 40. Ustekinumab demonstrated significant and lasting improvements in patient outcomes. At week 24, 35.6% of patients previously treated with ≥1 TNF and 54.4% of TNFi naïve patients achieved ACR20, versus 14.5% for placebo (**Figure 1**).

Furthermore, in a prospective observational study,^26^ 65 patients initiating ustekinumab therapy were evaluated. Of these, 80% had previously failed up to four TNFi, while 20% were biologic-naïve. Ustekinumab demonstrated clinical effectiveness, with 34% of patients achieving MDA at 24 months. Significant improvements were observed in joint outcomes, as the mean TJC decreased from 8.6 ± 7.5 at baseline to 2.7 ± 4.4 at week 24, and the SJC decreased from 2.7 ± 3.6 to 0.9 ± 2.1 (p < 0.0001 for both comparisons).

### TNFi to guselkumab

The COSMOS trial, a phase IIIb randomised, double-blind study,^27^ assessed guselkumab in 285 patients who had shown an inadequate response to prior TNFi. Participants were allocated in a 2:1 ratio to receive subcutaneous guselkumab 100 mg or placebo at weeks 0 and 4, followed by dosing every 8 weeks up to week 44. At week 24, within the primary analysis set, 44.4% of patients receiving guselkumab achieved an ACR20 response compared with 19.8% in the placebo group (95% CI: 14.1–35.2) (**Figure 1**).

### TNFi to secukinumab

A phase III, randomised, double-blind, placebo-controlled trial investigated the efficacy and safety of secukinumab, in adults with PsA.^28^ Patients were stratified based on prior TNFi therapy (anti-TNF-naïve or anti-TNF inadequate responders [IR]) and randomized in a 1:1:1:1 ratio. They received either placebo or secukinumab at doses of 75 mg, 150 mg, or 300 mg, once weekly from baseline and then every 4 weeks from week 4. At week 24, the proportion of patients achieving an ACR20 response was higher with secukinumab compared to placebo in both anti-TNF-naïve and anti-TNFIR populations, with the magnitude of response being higher in the anti–TNF naïve population. In TNFi-naïve patients, ACR20 responses were achieved by 58.2% of those receiving secukinumab 300 mg (p=0.004), 63.5% with 150 mg (p<0.0001), and 36.9% with 75 mg (p=0.0075), compared with 15.9% in the placebo group. Among patients with an inadequate response to TNFi, the corresponding response rates were 45.5% for the 300 mg dose (p=0.0077), 29.7% for 150 mg (p=0.1216), and 14.7% for 75 mg (p=0.9639), versus 14.3% with placebo (**Figure 1**).

### TNFi to ixekizumab

A double-blind, multicentre, randomised SPIRIT-P2 phase III trial^29^ demonstrated that ixekizumab is effective in reducing signs and symptoms in patients with PsA who did not respond adequately to anti-TNFi. Following an initial 160 mg dose, patients received either 80 mg every two or four weeks. At week 24, a higher proportion of patients attained ACR20 with ixekizumab every 4 weeks [53% vs placebo 33.8% (95% CI: 22.4–45.2)] and ixekizumab every 2 weeks [48% patients; 28.5% (95% CI: 17.1–39.8)] than did patients with placebo (**Figure 1**).

In another randomised, double-blind, placebo-controlled phase 3 study,^30^ Kirkham et al. assessed the efficacy of ixekizumab in patients who had an inadequate response to one or two TNF inhibitors. Patients were randomly assigned to receive a 160-mg starting dose of ixekizumab followed by 80 mg every 2 weeks (IXE Q2W), 80 mg every 4 weeks (IXE Q4W), or placebo through week 24. In the IXE Q4W group, 34% of patients with an inadequate response to one TNF inhibitor and 37% of those with an inadequate response to two TNF inhibitors achieved ACR50 at week 24.

Finally, an observational cohort study in Denmark^31^ explored the effectiveness of ixekizumab in 478 PsA patients, 91% of whom were previously treated with other biologic agents. The 24-month retention rates were 51% (95% CI: 46%–57%), while patients with prior Il-17i treatment had an increased risk of withdrawal of ixekizumab (HR: 2.38, 95% CI: 1.79–3.15).

### TNFi to bimekizumab

In a randomised, double-blind, placebo-controlled, phase 3 trial (BE COMPLETE),^32^ bimekizumab was compared with placebo over 16 weeks in patients with PsA or PsO who had an inadequate response to anti-TNF inhibitors. A total of 400 patients were randomly assigned to receive bimekizumab 160 mg every 4 weeks or placebo. The primary endpoint was achievement of ACR50 response at week 16. Results showed that 43% of patients receiving bimekizumab achieved ACR50, compared to 7% in the placebo group (p<0.0001). ACR20 scores at week 16 were 67.0% vs 15.8%, respectively (**Figure 1**). Additionally, 44% of bimekizumab-treated patients reached MDA versus 6% with placebo (p<0.0001).

Another retrospective, multicentre study conducted between January 2023 and February 2025 in two Italian centres,^33^ studied the effectiveness of bimekizumab in predominantly Difficult-to-treat (D2T) patients with PsA. Among the 40 patients included, the median number of previously failed b/tsDMARDs was 3.0 (2.0–3.3), and the median number of failed mechanisms of action was 2.0 (1.8–3.0). Notably, the median DAPSA score decreased significantly from 22.9 (17.9–28.7) at baseline to 10.7 (6.9–13.6) at week 12, and further to 6.0 (3.1–12.8) at week 24 (p < 0.001 for both). Median SJC declined from 3.0 (0.8–5.3) at baseline to 0.0 (0.0–2.0) at week 12 and maintained at week 24 (p < 0.001). TJC also showed significant improvement, dropping from 4.5 (3.0–7.3) at baseline to 2.0 (0.0–2.0) at week 12, and to 1.0 (0.0–2.0) at week 24 (p < 0.001).

### TNFi to JAKi

In a 6-month, randomised, double-blind, placebo-controlled phase 3 trial,^34^ 395 TNFi-IR patients were assigned in a 2:2:1:1 ratio to receive either 5 mg or 10 mg of tofacitinib twice daily, or placebo with a switch to active treatment at 3 months. At 3 months, the rates of ACR20 response were 50% with the 5-mg dose of tofacitinib and 47% with the 10-mg dose, compared to 24% with placebo (p<0.001 for both comparisons) (**Figure 1**).

In another 24-week, randomised, placebo-controlled, double-blind phase 3 trial, (35) 642 TNF-IR patients were assigned (2:2:1:1) to once-daily upadacitinib 15 mg, upadacitinib 30 mg, placebo with switch to 15 mg at week 24, or placebo with switch to 30 mg at week 24. The primary endpoint was the proportion of patients achieving an ACR20 response at week 12. At that time, 56.9% and 63.8% of patients receiving upadacitinib 15 mg and 30 mg, respectively, achieved ACR20 versus 24.1% with placebo (p<0.001 for both comparisons). At week 24, ACR20 responses were maintained in 59.2% of the 15 mg group and 61.5% of the 30 mg group, versus 20.3% with placebo (p<0.05) (Figure-1).

## CYCLING VS SWAPPING IN PSA

Researchers have also investigated whether the one strategy (i.e. cycling between drugs of the same mechanism of action [MOA]) is more efficient than the other (swapping to drugs with a different MOA).

A quasi-experimental study, conducted in the Netherlands by van Es et al.,^36^ exploiting the change in the local therapeutic protocols, examined the 3-year drug retention rate in 406 patients who received TNFi or IL-17i after failure of a first TNFi. No significant differences were found between the two groups (HR: 1.17, 95% CI: 0.87–1.58) apart from male patients which showed higher risk for drug discontinuation for swapping compared to cycling (HR: 1.64, 95% CI: 1.03–2.60).

Furthermore, data from a national registry in Portugal, including in total 439 individuals,^37^ suggest that retention rate was similar in patients who cycled after failure of a first TNFi, compared to those who swapped to secukinumab or ustekinumab. In fact, the retention rates after 6, 12, and 24 months were 72%/66%/59% and 77%/66%/59% in the cycling group and in the swapping group, respectively.

In another monocentric study by Ariani et al.,^38^ obtaining data from medical records, the retention rate of the drugs (biologic DMARDs, tofacitinib, apremilast) at 18 months was examined. Swapping (most of the patients in this group were treated with IL-17i) compared to cycling (most of the people in this group were treated with TNFi) did not display significant differences (HR: 0.95, 95% CI: 0.52–1.74).

Contrary to the results of the aforementioned studies, in a monocentric retrospective study (n=122) by Lumetti et al.,^39^ comparing cycling vs. swapping (TNFi to IL17i or vice versa) strategies in PsA patients, it was shown that swapping from TNFi to IL17i showed significantly better retention than cycling. In Cox regression models, the authors found that among others, swapping strategy affected positively (HR: 0.53, 95% CI: 0.31–0.89), the retention rate.

Drawing the main strands together, one could suggest that with the data gathered so far, cycling vs swapping strategies appear to have similar effects, whereas there might be some differences in specific patients’ subgroups (e.g. depending on gender). Apparently, the field for future studies examining this matter is open.

## CYCLING IN AXSPA

### TNF inhibitors

Although TNFi are highly effective in the treatment of AxSpA, if a patient fails to achieve adequate response during the first treatment course, cycling to a second TNFi seems to be a valid strategy. However, treatment effectiveness after cycling tends to be reduced.^40^

In RHAPSODY study,^41^ 1250 AS patients were enrolled. 924 of them had never received TNFi therapy and 326 had received at least one TNFi (infliximab, etanercept, or both -not concurrently). Adalimumab was administered for a 12-week period. ASAS40 response rates were 59.3% for TNFi naïve and 37.7% for patients with a prior TNFi therapy. In addition, BASDAI 50 was achieved in 63.0% of naïve patients vs 40.8% of AS patients who had already received prior anti-TNF treatment.

An observational multicentre study from Norway,^42^ assessed the effectiveness of cycling to a second TNFi; 514 patients with AS who were treated with their first TNFi were included (of those 77 cycled to a second TNFi). ASAS40 response was achieved by 38% of non-cyclers vs 30% of cyclers at 3 months; BASDAI50 was achieved by 49% vs 25% respectively, showing that the 3-month disease activity was higher for cyclers than non-cyclers.

In another multicentre retrospective observational study of 522 AxSpA patients,^43^ retention times of different subcutaneous TNFi were examined. Patients treated with first-line anti-TNF treatment had markedly longer median drug retention (48 months) compared with second-line (23 months) and ≥third-line therapy (29 months) (p=0.0004). Of note, Golimumab demonstrated a significantly higher overall retention compared with adalimumab, etanercept, and certolizumab (HR: 0.64, 95% CI: 0.43–0.97).

Ciurea et al. suggested also that the reason for discontinuation of the first TNFi could impact the effectiveness of a second TNFi.^44^ Specifically, 632 AxSpA patients who cycled to a second TNFi were studied. The patients discontinued the first TNFi due to primary lack of response (PLR) (23.1 %), secondary lack of response (SLR) (42.7 %), Adverse events AEs (19.8%) and other reasons (14.4%). Moderate disease activity (ASDAS- Erythrocyte Sedimentation Rate [ESR] <2.1) was achieved by 11 % and 39 % of patients in the PLR and SLR groups, respectively(p=0.01). ASDAS-ESR inactive disease state was observed in only 4% of patients after previous PLR in comparison to 22 % after SLR, concluded that the effectiveness of a second TNFi is reduced in patients with PLR to the first TNFi compared to SLR.

### IL-17 and JAK inhibitors

Data on IL-17 inhibitor cycling and in JAKi in AxSpA are limited, as there are no large studies currently available.

## SWAPPING IN AXSPA

### TNFi to secukinumab

**Figure 2.**
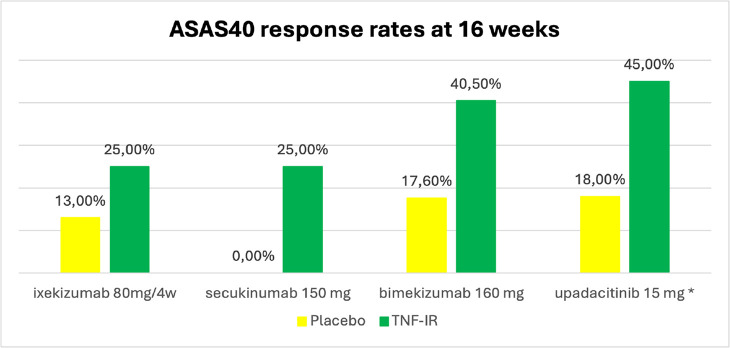
ASAS40 response rates of Ixekizumab, Secukinumab, Bimekizumab, and Upadacitinib in TNF-IR patients. *TNF-IR: TNF inadequate responders, r-: radiographic, Q4W: ixekizumab every 4 weeks, ^ week 14. Created by the authors using Microsoft Excel. Elements are authentic and not adapted from elsewhere. The comparisons shown are indirect with high risk of bias. The homogeneity of the population between studies cannot be assessed.

In the MEASURE 2 study, (45) 219 patients were randomly assigned to receive secukinumab (150 mg or 75 mg) vs placebo subcutaneously at baseline and at weeks 1, 2, and 3, and every 4 weeks thereafter. Of them, 61% were TNFi-naive and 39% were TNF-IR. Secukinumab 150 mg demonstrated superior ASAS40 improvements vs placebo at week 16 in both TNFi-naïve (19% vs 8%) and TNF-IR patients (25.0% vs 0%; p<0.05) respectively (**Figure 2**).

### TNFi to ixekizumab

The results of COAST-W study,^46^ provided insights into the efficacy of ixekizumab in patients with active radiographic axSpA, who were TNFi experienced. Patients were randomly assigned in a 1:1:1 ratio to receive placebo, ixekizumab (IXE) every 2 weeks (Q2W), or ixekizumab (IXE) every 4 weeks (Q4W). At week 16, the ASAS40 response rates were: 25% for IXE Q4W (**Figure 2**), 31% for IXE Q2W and for 13% placebo. Sustained improvement in disease activity scores, physical function and inflammatory markers were also detected for up to 52 weeks.

Real world data from DANBIO registry of 231 AxSpA patients, who started treatment with ixekizumab were recently reported.^47^ Almost all patients (96%) had previously received TNFi and 36% of them had been treated with another IL-17i. The 24-month treatment retention rates of ixekizumab were high (≈40%). Prior IL-17i use was linked to increased drug’s withdrawal rates (HR: 1.48, 95% CI: 1.01–2.17). At 6 months, 24% of patients had ASDAS-low disease activity and 5% were in ASDAS-remission. The improvement in all disease outcomes suggest that ixekizumab may be an effective option for patients with multiple b/tsDMARD failures.

### TNFi/IL-17i to JAKi

Results from SELECT-AXIS 2 study were presented,^48^ showing the efficacy and safety of upadacitinib in patients with active AS who had an inadequate response to one or two biologic DMARDs (TNFi or IL-17i). 420 AS patients were assigned in a 1:1 ratio to receive double-blind oral upadacitinib 15 mg once daily or placebo. At Week 14, a higher proportion of patients receiving upadacitinib achieved the primary endpoint of ASAS40 compared with placebo (45% vs 18%; p<0.0001) with a treatment difference of 26% (95% CI: 18%–35%). (**Figure 2**)

### TNFi to Bimekizumab

In BE MOBILE 2, (49) 332 radiographic (r-) AxSpA patients were randomized. Of them, 211 received bimekizumab and 111 placebo. At week 16, a higher proportion of TNFi-IR patients in the r-axSpA group achieved an ASAS40 with bimekizumab compared to placebo (40.5% vs 17.6%) (**Figure 2**), proving that bimekizumab is effective in axSpA patients irrespective of prior TNFi exposure.

## CYCLING VS SWAPPING IN AXSPA

In the above-mentioned quasi-experimental study, conducted in the Netherlands,^36^ it was found that among 335 axSpA patients (270 followed a cycling strategy and 65 a swapping strategy), risk for drug discontinuation (HR: 1.46, 95% CI: 1.03–2.07) was increased in the swap group compared to that seen in the cycle group. Min et al.,^50^ studied the drug retention rate and the efficacy of an alternative TNFi vs secukinumab in 124 AS patients of the Korean College of Rheumatology Biologics (KOBIO) registry, who previously experienced TNFi failure (46% were primary non-responders and 54% were secondary non- responders). Of them, 83 switched to an alternative TNFi and 41 altered to secukinumab. Cox regression analyses were performed to evaluate the frequency of drug discontinuation (secukinumab vs TNFi) after 1 year. In primary non- responders the HR for discontinuation of the treatment was non-statistically significant [(HR: 1.20, 95% CI: 0.32–4.51) and (HR: 0.42, 95%: CI 0.08–2.17), respectively]. Nevertheless, among secondary non-responders, secukinumab was associated with higher HR for drug discontinuation (HR: 3.77, 95% CI: 1.03–13.7) and these patients were less likely to achieve significant clinical response (BASDAI50 or ASDAS Major Improvement [MI]) than patients in the alternative TNFi group.

On the other hand, Micheroli et al.,^51^ reported a real - life observational cohort study of axSpA patients, in which 106 patients initiated secukinumab and 248 patients changed to an alternative TNFi after ≥1 prior TNFi failure. Notably, more patients of secukinumab group had received at least two different TNFi (76.4% vs 40.1% respectively). The results demonstrated comparable survival of secukinumab vs further TNFi (HR: 1.14, 95% CI: 0.78–1.68 and HR: 1.16, 95% CI: 0.79–1.71 in PS-based and multiple-adjusted models, respectively). BASDAI50 responses at 1 year also did not differ significantly (although the CI were broad) between these two biologic classes.

Oh Chan Kwon et al.,^52^ analysed factors influencing treatment retention of an alternative TNFi and secukinumab after switching from the first TNFi. In 78 patients with AS, researchers observed that higher C-reactive protein (CRP) levels at the time of switching (from TNFi to alternative TNFi) was linked to reduced risk of discontinuation of the second TNFi (adjusted HR: 0.93, 95% CI: 0.87–0.99). Furthermore, the primary failure of the first TNFi was correlated with increased risk (adjusted HR: 5.20, 95% CI: 1.91–14.11) of discontinuation of the second TNFi. In the case of secukinumab, (but not of the alternative TNFi) current smoking (p = 0.029) and presence of syndesmophytes (p = 0.019) at baseline were related to higher risk of treatment withdrawal.

In a real-world study of 557 axSpA patients,^53^ researchers assessed the efficacy of switching from a first TNFi to Upadacitinib (UPA) or to another TNFi or to an IL-17i; those who switched to UPA experienced significantly less pain (≥ 50%) in comparison to patients who cycled to TNFi (96.3% vs 72.3%; p = 0.024) or swapped to IL-17i (90.9% vs 74.0%; p =0.004). In addition, fewer affected joints were observed in the TNFi-UPA group than in the TNFi-IL-17i (93.1% vs 72.6%; p = 0.002) or TNFi-TNFi patients (100.0% vs 79.2%; p < 0.001).

All in all, although the evidence is not robust, it seems that after TNFi failure, swap to a different MOA is not superior to cycling, especially for secondary non-responders. It is still to be defined whether there are differences among bDMARDs. Although there is some preliminary evidence, the identification of risk factors that would affect the one or the other strategy warrants further research.

## CURRENT PERSPECTIVES

The management of PsA and AxSpA remains challenging, as many individuals need multiple treatment switches during their treatment. In this narrative review, we examined the current literature to determine whether one strategy is superior to the other.

Cycling between TNFi agents has proven to be effective in the management of PsA. However, multiple studies have demonstrated that the therapeutic response significantly declines with each TNFi, being lower for the second or third TNFi compared to the first TNF.^15,19^ Cycling between IL-17 inhibitors has also shown promising results. Notably, cycling from secukinumab to ixekizumab has proven to be an effective strategy. Nevertheless, an important question remains as to whether dual inhibition of IL-17A and IL-17F provides superior outcomes compared to selective inhibition of IL-17A alone. Interestingly, according to an observational cohort study in Denmark,^31^ patients with prior Il-17i treatment had an increased risk of withdrawal of ixekizumab compared to IL-17 naïve patients. This could mean that cycling between IL17i may have a similar effect with cycling between TNFi, with therapeutic responses declining with each IL-17i. However, further research is needed, as evidence is still scarce. IL-23 inhibitor cycling in PsA remains largely unexplored, data in psoriasis encourage IL-23 and IL-12/23 cycling.^24^

Swapping to treatment options with different MOA also appears to be a valid strategy, supported by multiple randomized controlled trials (RCTs). Swapping from TNFi to ustekinumab, guselkumab, secukinumab, ixekizumab, bimekizumab, or JAK inhibitors has been associated with clinical improvement, achievement of ACR20/50 responses and MDA, as well as DAPSA reductions. There is no hard evidence whether one drug is better than the other, upon failure of TNFi. However, bimekizumab seems to have better results so far (with all the limitations that an indirect comparison has). Data regarding the opposite direction, e.g. swapping from IL-17i to TNFi, are very limited.^39^

When directly comparing cycling versus swapping, cycling vs swapping strategies appear to have similar effects. Most studies suggest similar drug retention rates between the two strategies, while others,^39^ suggest better outcomes with swapping. Interestingly, gender differences have been suggested, with male patients showing higher discontinuation rates when swapping compared to cycling, though this requires further validation.

In the case of AxSpA, failure of a first TNFi does not preclude the response to another. However, cycling to another TNFi is associated with diminished treatment responses and/or decreased drug survival among these patients.^54^ Data from 12 European registries were collected to investigate effectiveness of the second and third TNFi-series in 8254 and 2939 patients respectively.^55^ Twelve-month retention rates did not differ between patients who discontinued the prior TNFi due to adverse events (AE) versus lack of response, both for the second (68% vs 67%) and the third TNFi (both 68%). However, for the second TNFi, retention was lower among primary non-responders compared with secondary non-responders (58% vs 71%, *P* < 0.001). ASDAS inactive disease at 6 months was achieved in 23% of patients receiving a second TNFi in comparison to 16% of those receiving a third one, emphasizing that effectiveness decreases with each TNFi cycling.

The same phenomenon seems to hold true for IL-17 inhibitors. A real-world study of 1860 patients^56^ analysed the effectiveness of secukinumab in AxSpA patients at 6 and 12 months. It was revealed that retention at 6 and 12 months was 82% and 72%, respectively, with significant differences observed according to the number of prior b/tsDMARDs (p ≤ 0.001). Decreasing drug retention rates with increasing previous b/tsDMARD use were observed (6-/12-month: b/tsDMARD naïve: 90%/84%, 1 prior b/tsDMARD: 83%/73%, ≥2 prior b/tsDMARDs: 78%/66%); HR 1.78, **95% CI 1.29–2.47** for 1 prior, HR 2.33, 95% CI 1.74–3.11 for ≥2 prior.

One must interpret the results of the abovementioned studies with caution as most of them are retrospective observational and so biases are inevitable. Furthermore, our review was narrative and not systematic and thus it is possible that studies might have been missed. Moreover, data about the reason of treatment failure were not always reported; there is some evidence that there are differences between primary and secondary failure. On the other hand, to the best of our knowledge, this is the first manuscript reviewing this topic in both PsA and SpA, identifying the need for future study in the field. A third strategy, that of combining two different b/tsDMARDs might come also in the rheumatologist’s arsenal soon.^57^

Furthermore, it should be noted that the comparisons illustrated in the figures are indirect. The homogeneity of the population between studies cannot be assessed. Therefore, these comparisons have a high risk of bias. Previous publications in the Mediterranean Journal of Rheumatology have provided overviews of the therapeutic approaches in PsA and AxSpA.^58–60^ However, none of the aforementioned studies has directly examined the comparison between cycling and swapping in SpA. Our review advances the discussion by bridging the gap between prior MJR theoretical knowledge and everyday clinical decision-making, offering practical insights into the selection of agents with the same or a different MOA.

## CONCLUSION

To conclude, in PsA and AxSpA, both cycling and swapping strategies appear to be equally successful in general. Characteristics, like gender, might affect the efficacy of the one versus the other strategy but research in the field is warranted.
